# The Role of Internal Carotid Artery Stent in the Management of Skull Base Paragangliomas

**DOI:** 10.3390/cancers16132461

**Published:** 2024-07-05

**Authors:** Riccardo Di Micco, Rolf Benedikt Salcher, Friedrich Götz, Omar Abu Fares, Thomas Lenarz

**Affiliations:** 1 Department of Otolaryngology, Hannover Medical School, 30625 Hannover, Germany; 2 Department of Neuroradiology, Hannover Medical School, 30625 Hannover, Germany

**Keywords:** paraganglioma, internal carotid artery, stent, preoperative stenting

## Abstract

**Simple Summary:**

In lateral skull base surgery, one of the greatest challenges remains the internal carotid artery. Paragangliomas can encase and finally invade the intrapetrosal carotid artery, making preservation of the vessel and of sufficient cerebral blood supply challenging. The use of intracranial endovascular stents makes the preoperative reinforcement of the arterial wall feasible, allowing the surgeon to deliberately manipulate the vessel and perform a safe tumor dissection from the stented artery without sacrificing it. It represents a valid alternative to more aggressive preoperative vascular treatments worth considering in cases of extensive vascular encasement of the internal carotid artery to increase surgical safety and radicality.

**Abstract:**

**Background:** After two decades from its introduction in the lateral skull base paraganglioma surgery, the indications and results of preoperative internal carotid artery stenting should be critically assessed. **Materials and Methods:** Monocentric retrospective study on 26 patients affected by head and neck paragangliomas (19 tympanojugular paragangliomas, 4 carotid body paragangliomas, 3 vagal paragangliomas) preoperatively treated with internal carotid artery stents between 2008 and 2023. The preoperative findings, the intraoperative complications and the final surgical results were analyzed. **Results:** The stent complication rate was less than 3.1%. Self-expanding highly flexible intracranial nitinol stents were applied. In all cases, it was possible to completely mobilize the internal carotid artery and perform a vascular dissection of the tumor. Gross total tumor resection was possible in 85% of cases. The median follow up was 7.83 y (SD +/− 3.93 y). No local recurrence was observed. **Conclusions:** The preoperative vascular stent facilitates tumor dissection from the internal carotid artery without risk of vascular damage, helping the surgeon to achieve surgical radicality. The vascular stent is indicated in the case of revision surgeries, circumferential involvement of the vessel and in cases with non-insufficient intracerebral crossflow. Procedural complications, temporary antiplatelet therapy and delay of surgery are the limitations of the procedure.

## 1. Introduction

In the surgery of paragangliomas involving the lateral skull base, one of the major challenges remains the intraoperative control of the internal carotid artery (ICA), which can be engulfed by the tumor mass [1]. The hypervascular architecture of paragangliomas and the intimate relationship with the vessel pose major challenges during the dissection from the artery [2], especially in its more fragile horizontal intrapetrosal segment. The genu of the ICA is also the most common localization for recurrent disease in tympanojugular paraganglioma, possibly due to previous incomplete surgeries [3]. Paragangliomas of the jugular foramen or the vagus can encase and finally invade the intrapetrosal carotid artery, making preservation of the vessel and of cerebral blood supply challenging. This is true for cases with insufficient cerebral crossflow between the two ICAs. With the introduction of intracranial endovascular stents [4], reinforcement of the arterial wall has become feasible as a preoperative strategy. The stent mesh is covered by neointima within 4–6 weeks [5], creating an additional arterial wall which allows the surgeon to deliberately manipulate the vessel and perform a subadventitial dissection of the tumor even in the horizontal segment of the ICA. After almost two decades from its introduction in lateral skull base surgery, the indications and results of carotid stenting should be critically assessed, as compared to alternative strategies, such as permanent balloon occlusion (PBO) and intracerebral shunting.

## 2. Materials and Methods

Between 2008 and 2023, 112 patients were treated for skull base and neck paragangliomas in our institution. Of them, we identified thirty-two patients with advanced cases scheduled for surgical resection who required reinforcement of the vascular wall according to our criteria in order to reduce the intraoperative risk of vascular damage. Of them, 26 (8 males, 18 females) underwent subsequent tumor surgery and were enrolled in a monocentric retrospective study (Table 1). Inclusion criteria were defined paraganglioma in the histological section, extensive vascular involvement in the preoperative imaging, ICA stenting followed by surgery, complete preoperative work-up and ongoing follow-up. Exclusion criteria were incomplete data, ICA stenting not followed by extirpation surgery and patient lost to the follow-up.

**Figure 1:**
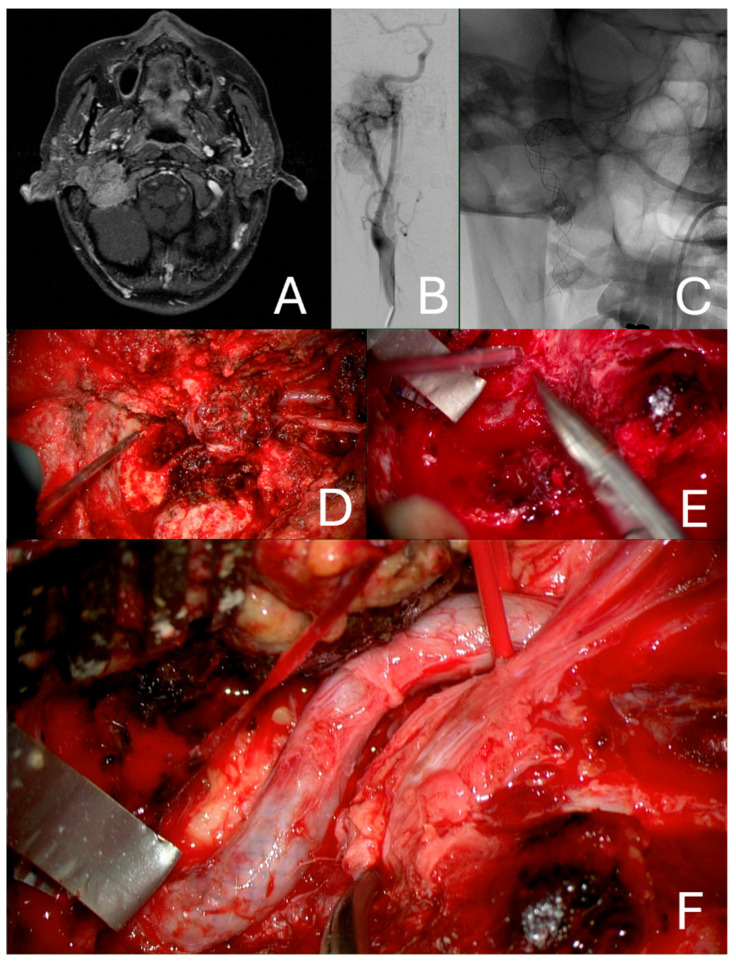
Radiographic representation of a C3De2 tympanojugular paraganglioma of the right side. (**A**) Angiographic presentation showing involvement of the genu and horizontal segment of the ICA (**B**). Position of a LEO Stent (**C**). Initial stage of tumor removal of the tumor through an Infratemporal fossa approach Type A. The tumor extends along the horizontal segment of the ICA (**D**). Identification of the cleavage plane using the stent mesh as a reference to perform sharp tumor resection. (**E**) Final stage of the subadventitial dissection of the ICA, the vessel can be safely manipulated to reach the medial wall of the carotid canal and the anterior temporal bone, allowing for complete tumor removal (**F**).

All patients underwent preoperative magnet resonance imaging (MRI) and high-resolution computer tomography (CT) together with a digital subtraction angiography (Figure 1A–C) to evaluate the tumor vascularization and extension and the intracranial crossflow, which was evaluated with the Mata’s test for the anterior communicant artery and the Alcock’s test for the posterior one. Based on radiomorphological studies, the tumors were classified according to the Fisch classification in the case of Tympanojugular paragangliomas [6], or the Shamblin classification for carotid body paragangliomas [7]. In our institution, we indicate endovascular ICA stenting in the case of multifocal disease on the contralateral side [8], stenosis of the vessel, presence of feeder tumor vessel from the ICA, tumor encasing of the ICA major of 270° in any of its segments, involvement of the horizontal of the intracranial part of the ICA, insufficient intracerebral crossflow in the circle of Willis, intolerance to the ballon occlusion test or monolateral ICA. Patients who did not qualify for stenting due to excessive kinking or stenosis of the vessel received a permanent ballon occlusion if tolerated. The stenting procedure was performed under general anesthesia three months before elective tumor surgery and all patients were subsequently followed up in our institution. As in all cases, the objective was to reinforce the vascular walls in order to preserve the ICA, and permanent balloon occlusion was always disfavored if possible.

The stenting procedure was performed under general anesthesia and self-expanding nitinol stents were applied: LEO Stents (Balt Extrusion, Montmorency, France), LVIS EVO Stent (Microvention Terumo, Aliso Vejo, CA, USA), Pipeline vantage Flow Diverter (Medtronic, Minneapolis, MN, USA) and Wallstent (Boston Scientific, Marlborough, MA, USA). On the morning of the procedure, all patients received a loading dose of 100 mg acetylsalicylic acid and 300 mg clopidogrel to reduce the risk of thromboembolic events. Starting the day after the procedure, patients received a maintenance regimen of 100 mg acetylsalicylic acid and 75 mg clopidogrel per day for three months. Afterwards, a monotherapy of 100 mg acetylsalicylic acid daily was indefinitely prescribed [9]. As one of the advantages of the stenting procedure is to reinforce the vascular wall so as to allow major manipulation of the vessel without risking tearing or shearing damages during tumor dissection, we strived to deploy the stent so as to cover 1 cm of tumor-free vessel wall both cranially and caudally. We oversized the stent diameter to achieve tight wall apposition. Due to tumor extension, usually more than one stent may be required to properly cover the entire area.

Three months after stenting, it was assumed that neointima had been developed and patients were scheduled for eradication surgery. The antiplatelet therapy was discontinued one week before surgery and resumed four days afterwards. Low molecular weight heparin was given during the intervening period.

**Figure 2:**
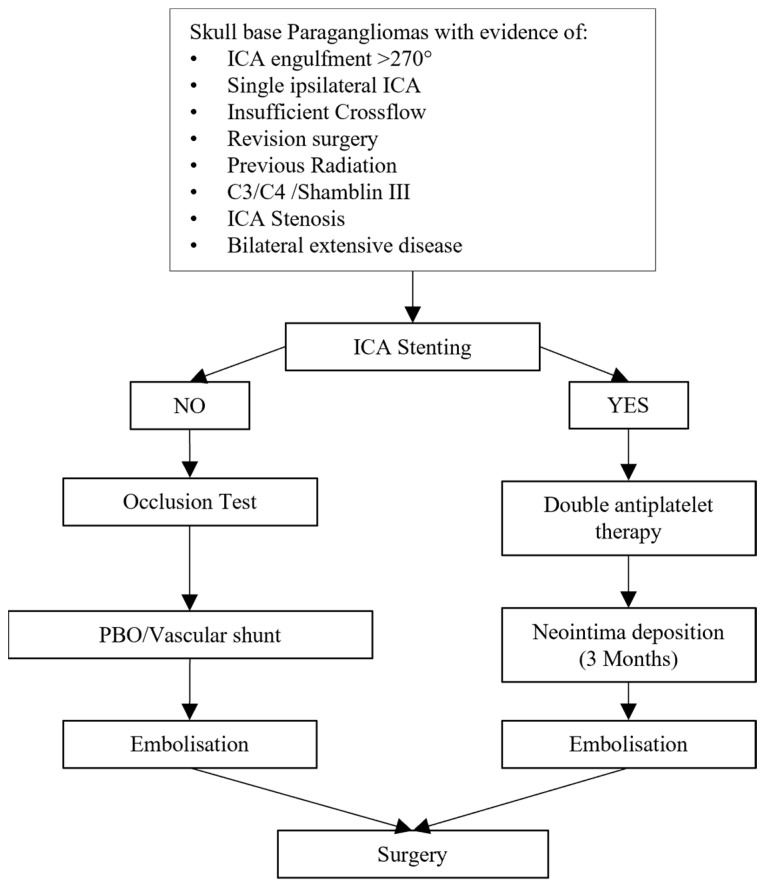
Preoperative workflow by paraganglioma with extensive ICA involvement.

In our center, all patients with C2–C4 tympanojugular paraganglioma, vagal paraganglioma and Shamblin III carotid body paraganglioma underwent a preoperative selective embolization of the tumor one to three days before surgery to reduce its vascularization (Figure 2).

In the case of tympanojugular paraganglioma or vagal paraganglioma, an infratemporal fossa approach type A was invariably applied. The surgery was also not staged in the case of large intradural components. For carotid body paraganglioma, a transcervical approach was preferred, with a transmastoid extension in case of involvement of the jugular bulb.

The surgical results were evaluated based on the intraoperative control of the ICA, extent of tumor resection and vascular and neural complications after surgery. All patients had a yearly follow up including serial MRI scan to detect tumor recurrence.

The ethics committee approved the protocol for using the patients’ data for this retrospective study (Project identification code 1897–2013). All patient data were anonymized and de-identified prior to the retrospective analysis.

## 3. Results

A total of 26 (8 males, 18 females) patients underwent ICA stenting and subsequent tumor surgery between 2008 and 2023. Specifically, nineteen presented a tympanojugular paraganglioma, three with a vagal paraganglioma and four with a carotid body paraganglioma (Table 1). The mean age at the time of surgery was 54.06 y (SD +/− 16.93 y).

In ten cases there was a history of previous surgery with subsequent tumor recurrence within one to 20 years, three patients had undergone previous radiotherapy without success. Fifteen tympanojugular paragangliomas (TJP) showed an involvement of the horizontal segment of the ICA with circumferential encasement of the vessel. The remaining four cases were characterized by non-sufficient cerebral crossflow. Two cases presented multifocal bilateral involvement.

The three vagal paragangliomas were advanced cases with encasement of the ICA and extensive cranial nerve involvement with vagus and glossopharyngeal palsy on the affected side.

All carotid body paraganglioma presented an extensive circumferential involvement of the ICA, qualifying as Shamblin III, in two cases there was extension into the temporal bone.

The stenting procedure was uneventful and well tolerated but in one case (3.1%) of TJP recurrence in a 61-year-old female with a history of two previous incomplete surgeries in other institutions. During the operation, an infiltration of the wall of the horizontal segment was detected and we decided due to absent crossflow in the angiography to discontinue the procedure, as it was not possible to guarantee radicality and vascular preservation. A flow diverter was implanted seven days afterwards, but the patient developed anisocoria within 24 h and contralateral hemiparesis. The MRI identified multiple infarctions in the thalamus and self-limited caudatus bleeding. The symptoms completely resolved within five days with conservative measures under constant double antiplatelet therapy. There were no permanent sequelae. Tumor removal was performed three months later with ICA preservation and no vascular complications.

In one case of tympanojugular paraganglioma, excessive stenosis prevented the positioning of a stent and we opted for a permanent ballon occlusion.

In all cases, the stent allowed dissection of the tumor from the vascular wall and a complete mobilization of the vessel to control the medial aspect of the carotid canal and the temporal bone without sacrificing the ICA (Figure 1D–F). In the case of involvement of the foramen lacerum, the tumor dissection was done towards the second vertical segment.

In one case of carotid body paraganglioma, the ICA was damaged at the level of the carotid bifurcation as a small hole developed between the frame of a LEO stent and required a vascular reconstruction with muscle.

In twenty-two cases treated with stent, total microsurgical tumor extirpation was achieved (85%). Subtotal extirpation was possible in four cases of tympanojugular paraganglioma with intradural extension. We experienced three cases of postoperative cerebrospinal fluid leak and one case of postoperative cerebellar bleeding in patients suffering from tympanojugular paraganglioma with extensive posterior fossa dural involvement. One patient died 14 days after surgery due to sudden pulmonary embolism unrelated to the surgical procedure.

The mean follow-up was 7.83 y (SD +/− 3.93 y), there was no clinical or radiological evidence of local recurrence or progression of the pathology. There was no evidence of stent-related complication in the long term, especially no evidence of stenosis, occlusion or aneurysm of the carotid artery.

## 4. Discussion

The treatment of skull base paragangliomas has evolved in recent decades along the three major axes of surgical excision [10,11], radiosurgery [12,13,14] and wait and scan [15,16]. Surgery should aim to eradicate the disease as radically as possible and acceptable in the specific case [17,18], but the risk of damage of the ICA poses the major limitation for radical tumor resection of skull base paragangliomas with a vascular involvement. Complete tumor dissection from the wall of the vessel might be impossible in cases of tumor infiltration of the media layer. On the other hand, it is usually possible to find a cleavage plane between the tumor and the arterial vascular wall under a microscope, however the procedure might lead to ICA rupture and subsequently also have lethal consequences in some patients [19]. This is especially true for the intrapetrosal genu and horizontal segment of the ICA due to a thinner medial layer [20]. The risk of damage grows exponentially and a vascular lesion in this region can be difficult to control using lateral skull base approaches [21].

A possible compromise is the partial resection of the tumor, counting on the slow growing rate of paragangliomas [15,22] and on less invasive subsequent adjuvant or salvage radiotherapy [23]. This treatment has shown its efficacy in lowering surgical risk and lower cranial nerve damage in extensive tumors [24]; nevertheless, there is the risk of simply postponing the problem as the patient can require subsequent revision surgery due to uncontrolled symptoms or tumor progression, adding even more challenges due to deposition of scar tissue. Consequently, it is necessary to implement preoperative procedures which allow radical tumor resection with a negligible risk of ICA damage, and increase radicality in first-line as much as in revision surgeries.

The previous benchmark was the permanent ballon occlusion technique [25,26], which has to sacrifice the affected ICA in order to achieve radical surgical excision of the tumor. However, this procedure has to be limited to patients with sufficient cerebral crossflow, otherwise it has the inherent risk of a stroke due to non-sufficient cerebral perfusion [27]. As paraganglioma could be multifocal and bilateral, it is also not possible in patients who have already lost the contralateral ICA in previous surgeries. The use of endovascular stents was initially proposed exactly to manage these cases [4].

An alternative to be considered is a vascular bypass [28]. Although many different techniques exist, it remains a major surgical operation with substantial risk of thromboembolism, shunt insufficiency, anastomosis blow-out and inherently connected morbidity and mortality. Considering the morbidity of the subsequent extensive lateral skull base surgery, this option must be critically assessed [9]. Staged surgical interventions are required, but it remains a possible, albeit complex, option in very selected cases.

The use of endovascular stents to reinforce the ICA wall before tympanojugular paraganglioma surgery has become a treatment option in various institutions [29,30]. The stenting procedure offers three practical advantages. First, it allows efficient preservation of the entire ICA, irrespective of tumor extension and inadequate contralateral circulation in the circle of Willis. The limitations of the permanent ballon occlusion and the risky and complex procedure of an ICA bypass are simply avoided. Secondly, the formation of a neointima on the stent within 4–6 weeks after implantation allows for a strengthening of the vessel, allowing for a more aggressive vascular dissection granting more protection from sharing and tearing damages. The external surface can be exposed during surgery, facilitating the identification of a safe cleavage plane from the tumor, which can be safely peeled off the vessel. Flow diverters offer even more security, as their fine mesh structure even prevents perforating damage, which can be an issue when using other self-expanding stents such as the LEO stent as in our case. Moreover, the strengthened vessel can be more transposed to control the medial aspect of the carotid canal and the anterior part of the temporal bone, allowing for bony decompression and tumor removal without risking vascular damage. A strengthened vessel is particularly helpful in the case of revision surgery or after irradiation, as scar tissue and post-irradiation tissue damage make the vascular walls even more fragile. Thirdly, the stenting procedure is comparatively safe and straightforward, although the risk of vascular events must not be underestimated [31]. We experienced one case (3.4%) of thalamus infarction correlated to stenting; however, it is worth noting that the stent procedure was performed only seven days after revision surgery; consequently, previous operative vascular manipulation could have increased the risk of thromboembolic events in the acute phase. Carotid angiography carries a risk of permanent neurological deficit of less than 1% [32]; however, the risk of stroke can increase to 4.5% during carotid stenting in cases of stenosis [33], comparable to our findings, as the paraganglioma may reduce the vascular lumen due to compression. However, the stenting procedure is less dangerous than the standard balloon occlusion test, where the intraprocedural stroke risk is as high as 16% [34]. Our case series is too small to determine low percentage risks, however possible cerebrovascular complications must be taken into consideration in the preoperative planning.

**Figure 3:**
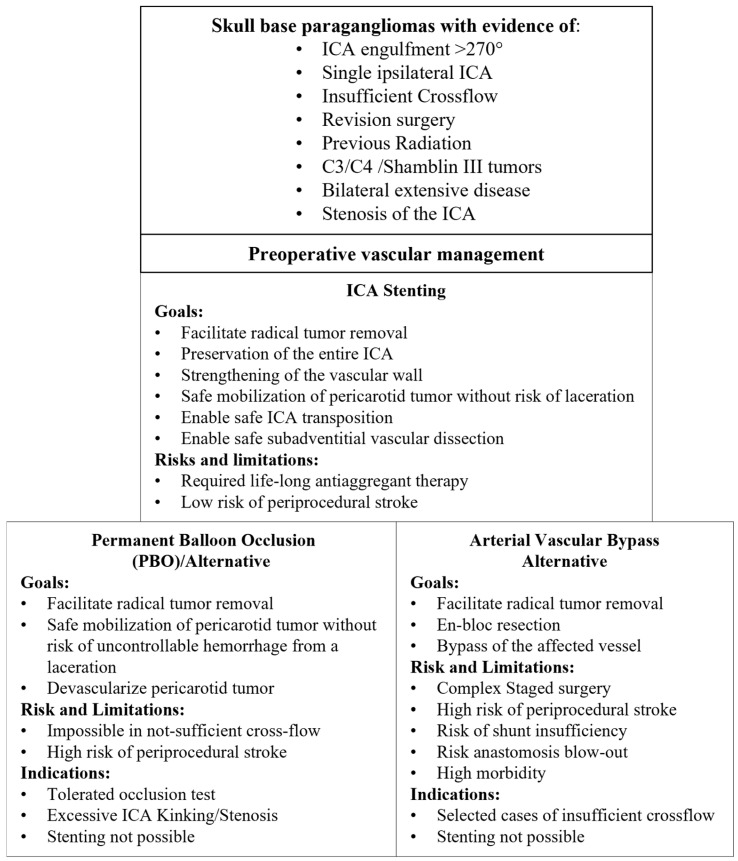
Proposed preoperative vascular management for skull base paragangliomas scheduled for surgery with extensive involvement of the ICA.

The patients only require a regimen of double antiplatelet therapy for three months to avoid stent-induced thrombosis. Afterwards, the daily intake of 100 mg acetylsalicylic acid suffices [35]. The main limitations remain the necessity of an additional angiography to place the stent and rescheduling the major surgery, which we suggest extending to three months to avoid unwanted drug-induced intraoperative bleeding. In our view, the current application of the permanent balloon occlusion remains limited to those cases where excessive kinking or stenosis of the ICA prevent stent placement (Figure 3).

Although preoperative stenting of the ICA in paraganglioma surgery remains an option up to the preference of the individual surgical team, it has shown to be a safe procedure that helps in avoiding vascular damage and improving the surgical radicality for the extradural part of the tumor [26]. The use of a stenting procedure does not reduce the necessity of a preoperative tumor embolization in extended tympanojugular paraganglioma [36,37,38], otherwise it could not be possible to achieve a sufficiently dry surgical field to visualize key neurovascular structures critical for reducing surgical morbidity and increasing the probability of gross tumor removal [17]. The same principles are appliable for vagal paraganglioma with ICA involvement [39].

In the case of carotid body tumors, the circumferential involvement of the internal and external carotid arteries could hamper the surgical removal and the use of intravascular stenting is of equal use to allow safe dissection of the tumor from the vascular wall in cases of total or subtotal encasement [40]. There is a reported reduction of subsequent tumor vascularization [41]. Our results are comparable with the available literature [42,43,44]. It is possible to achieve the same surgical results without stent application and even embolization [45]; however this is at the cost of a greater risk of vascular injury [46] and subtotal tumor removal.

## 5. Conclusions

The use of internal carotid artery stenting in the perioperative management of tympanojugular paragangliomas offers a valid and safe surgical strategy to improve vascular control, reduce risk of intraoperative major bleeding and increase radical tumor extirpation. The stenting procedure is standardized and safely appliable and improves surgical control of the intrapetrous segment of the ICA. Its use allows for safe mobilization of the vessel without incurring tearing or shearing damage of the external vascular walls, allowing for total tumor resection, even in advanced and complex cases. Although the application of the stenting comes down to local surgical expertise and practice, there is an undeniable increase in surgical safety in cases where a PBO or a bypass should otherwise be considered, allowing preservation of the vessel integrity. The application of preoperative stenting offers a way to increase safety, radicality and operability, allowing the surgeon to aggressively dissect the ICA with comparatively negligible risk of vascular damage. The risk of procedural complications, temporary double antiplatelet therapy and rescheduling of surgery are the main limitations of the procedure.

## Figures and Tables

**Table 1:** List of patients undergoing preoperative treatment of the ICA in paraganglioma surgery.

Tympanojugular Paraganglioma								
Patient n.	Age	Sex	C Class	Previous Therapy	Angiography	Encasement (MRI)	Stent	Surgical Removal
1	52	F	C2	Surgery 1 year before	Insufficient crossflow	270°	LVIS Stent x2	Total
2	63	F	C3	/	Kinking and Stenosis	360°	Impossible, PBO	Total
3	62	F	C2 Bilateral	Surgery 19 years before	Insufficient crossflow	270°	LEO Stent x2	Total
4	26	M	C4	Surgery 4 years before, Radiotherapy	Stenosis	270°	LEO Stent/Wallstent	Subtotal
5	29	M	C4	/	Stenosis	360°	LEO Stent x2	Subtotal
6	70	M	C2	Radiotherapy	Insufficient crossflow	180°	LEO Stent x2	Total
7	39	F	C3	/	Stenosis	270°	LEO Stent x2	Total
8	59	F	C3	Surgery 3 years before	/	270°	LEO Stent x2	Total
9	64	M	C3	Surgery 1 year before	Stenosis	360°	LEO Stent x2	Subtotal
10	30	F	C3	/	Stenosis	270°	LVIS Stent x2	Total
11	72	F	C3	/	/	180°	LEO Stent x2	Total
12	49	F	C3	/	/	360°	LEO Stent 2	Total
13	57	F	C2 bilateral	Surgery 2 years before, Radiotherapy	Insufficient crossflow	180°	Leo Stent/Wallstent	Total
14	75	F	C3	Surgery 9 years before	/	360°	LEO Stent/Wallstent	Total
15	30	F	C3	/	/	270°	Flow diverter	Total
16	59	M	C4	Surgery 20 years before	Stenosis	270°	LEO Stent/Wallstent	Subtotal
17	52	F	C3	/	/	270°	LEO Stent x2	Total
18	72	F	C3	/	/	180°	LEO Stent x2	Total
19	61	F	C2	Surgery 3 years before	Insufficient crossflow	270°	Flow diverter	Total
**Vagal Paraganglioma**								
**Patient n.**	**Age**	**Sex**	**C Class**	**Previous Therapy**	**Angiography**	**Encasement** **(MRI)**	**Stent**	**Surgical Removal**
1	68	F	C2	/	Stenosis	360°	LEO Stent x2	Total
2	37	F	C3	/	Stenosis	270°	LEO Stent x2	Total
3	35	M	C3	/	Stenosis	270°	LEO Stent x2	Total
**Carotid Body Paraganglioma**								
**Patient n.**	**Age**	**Sex**	**C Class**	**Previous Therapy**	**Angiography**	**Encasement** **(MRI)**	**Stent**	**Surgical Removal**
1	37	M	III with temporal infiltration	/	stenosis	360°	ICA Wallstent	Total
2	48	F	III	/	stenosis	360°	ECI Leo Stent/I ICA Wallstent	Total
3	47	M	III with temporal infiltration	/	/	360°	ECI Solitaire/ ICA Wallstent	Total
4	44	F	III	/	/	360°	ECI LEO Stent/ICA Wallstent	Total

## Data Availability

The data are not available die to ethical restrictions.
